# Successful surgical treatment of traumatic sternal fracture with extensive mediastinal abscess and concomitant mitral valve endocarditis: a case report

**DOI:** 10.1186/s13019-016-0507-y

**Published:** 2016-08-15

**Authors:** Hiroshi Munakata, Yu Murakami, Katsuhito Mabuni, Hiroyuki Tsuchiya, Moriaki Shinzato, Takehiro Umemura, Tadao Kugai

**Affiliations:** 1 Department of Cardiovascular Surgery, Okinawa Prefectural Nambu Medical Center and Children’s Medical Center, Haebaru, Japan; 2 Department of Emergency and Critical Care Medicine, Okinawa Prefectural Nambu Medical Center and Children’s Medical Center, Haebaru, Japan

**Keywords:** Traumatic sternal fracture, Mediastinitis, Endocarditis, Aggressive debridement, Successfully staged strategy

## Abstract

**Background:**

A traumatic sternal fracture with extensive mediastinal abscess and concomitant native valve endocarditis is an extremely rare but catastrophic situation.

**Case presentation:**

For 2 weeks, the co-infected patient was treated with aggressive debridement for the mediastinitis, change of vacuum-assisted closure therapy dressings, vegetectomy and valve repair through lower partial sternotomy, and delayed primary wound closure.

**Conclusions:**

To the best of our knowledge, this successful staged strategy has not been previously reported. We believe that our quick decision about repeated surgical interventions and preservation of the manubrium led to a favorable result.

## Background

A mediastinal abscess because of a primary traumatic sternal fracture is uncommon in healthy individuals [1, 2]. Additionally, native valve endocarditis with concomitant sternal mediastinitis is an extremely rare but catastrophic situation. To the best of our knowledge, this catastrophic situation has not been previously reported, nor has a successful strategy been employed to manage this situation; hence, we report a successful surgical strategy for this serious, co-infected case.

## Case presentation

The patient was an 87-year-old healthy female with only a medical history of hypertension. One month earlier, she experienced blunt chest trauma. During this period, general malaise and intermittent fever occurred.

Initially, the patient was admitted to another emergent hospital because of excruciating chest pain and severe dyspnea. Emergent computed tomographic (CT) scan demonstrated a transverse mid-body fracture of the sternum with surrounding fluid collection. The patient was transferred to our institution with a diagnosis of traumatic sternal fracture with mediastinal abscess.

**Fig. 1:**
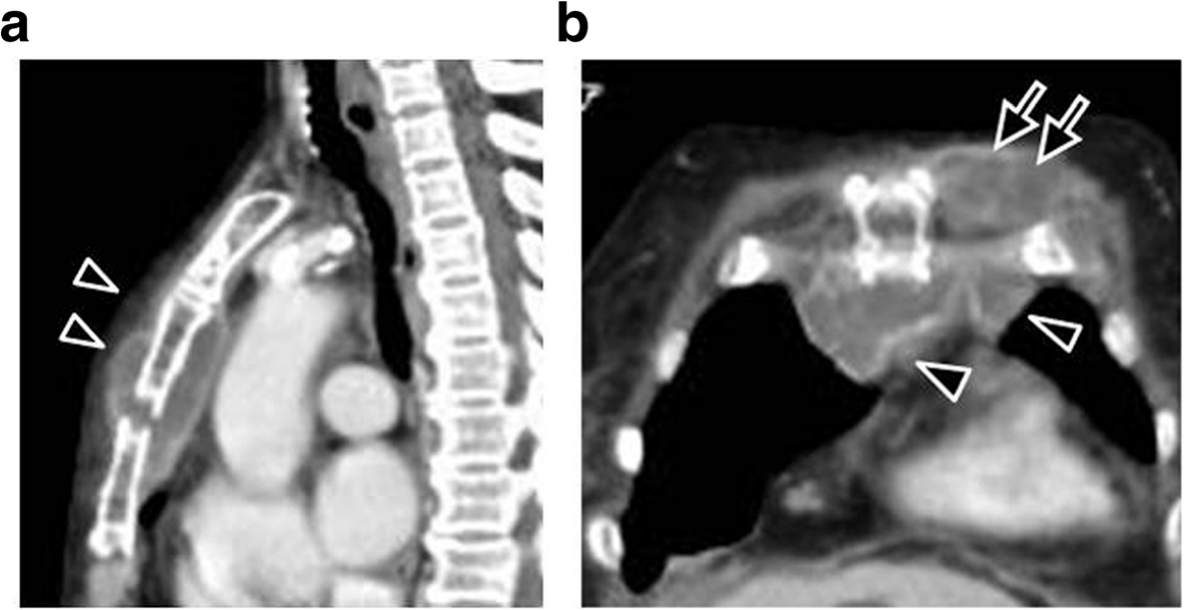
Contrast-enhanced computed tomography images 4 weeks after blunt chest trauma. **a** Sagittal image and **b** Axial image. The sternum was fractured and the mediastinal abscess can be seen anterior and posterior to the sternum (*arrowheads*) and extending to the left pectoralis muscles (*arrows*). Four weeks after traumatic accident

**Fig. 2:**
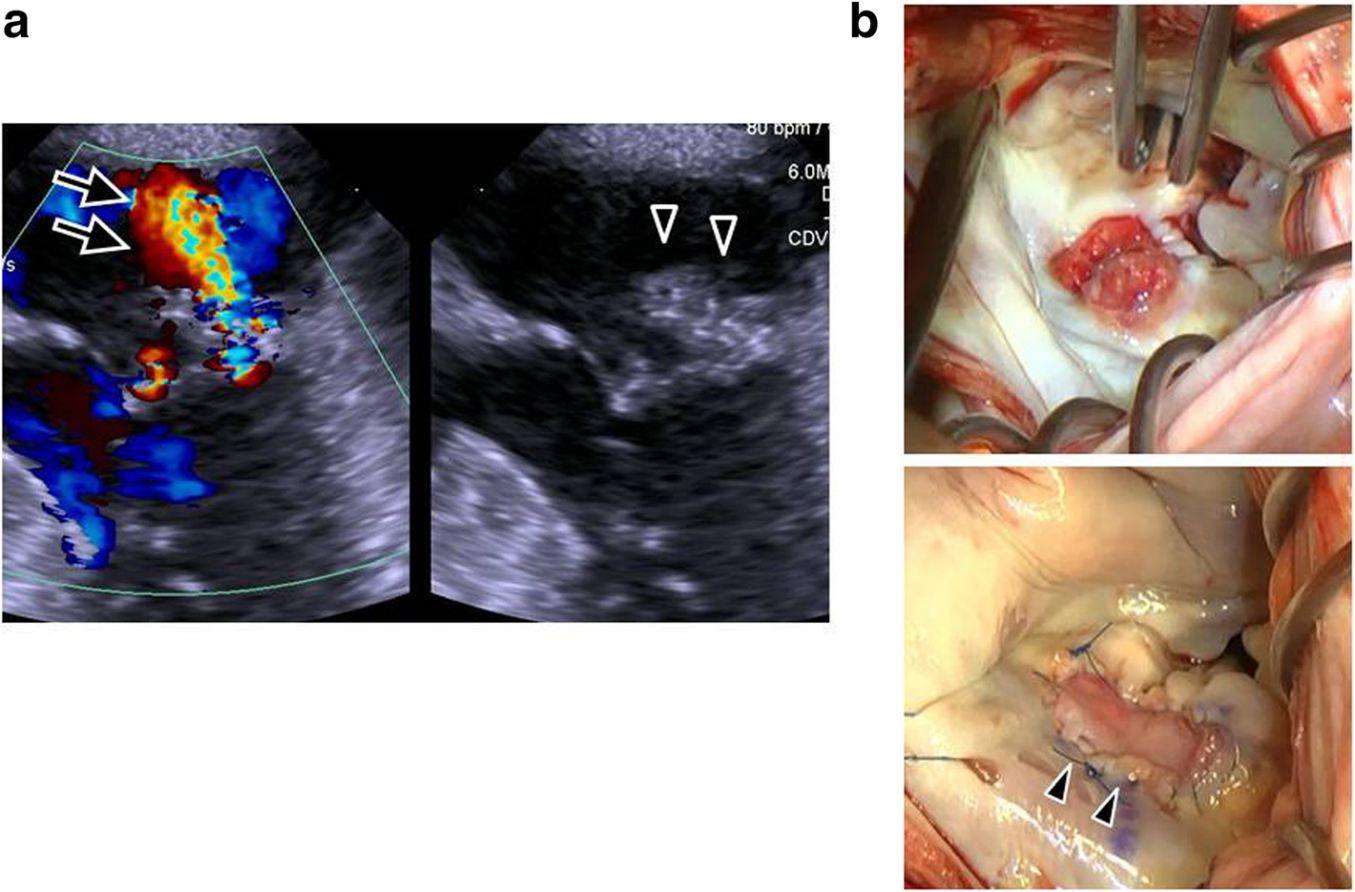
Concomitant mitral valve endocarditis. **a** Transesophageal echocardiography showed a large vegetation (15 × 17 mm) on the posterior mitral leaflet (*arrowheads*) and moderate insufficiency because of leaflet perforation (*arrows*). **b** Intraoperative mitral valve findings through the left atrial approach. *Upper*; A large vegetation was present on the middle posterior leaflet {P2 scallop}. *Lower*; Complete resection and repair of mitral valve using autologous pericardial patch (*arrowheads*). Concomitant mitral valve endocarditic

On admission to our institution, the patient had high grade fever. Initial laboratory data demonstrated a white blood cell (WBC) count of 25,100/uL and C-reactive protein (CRP) of 340 mg/L. Blood culture was positive for Gram-positive cocci in clusters and vancomycin was immediately started. A few days after admission, her inotropic requirement increased progressively as her hemodynamic status deteriorated. She was endotracheally intubated and follow up images showed progressive changes. The abscess could be seen extending to both the anterior and posterior aspects of the fractured sternum and to the left pectoralis muscles and subcutaneous tissues (Fig. 1a, b). Moreover, transesophageal echocardiography was done because of her bacteremia and it showed a large vegetation in the mitral valve with moderate insufficiency (Fig. 2a). We believed that definitive surgical treatment involving aggressive debridement, probably followed by vegetectomy, is the only effective method of saving our seriously co-infected patient with extensive mediastinitis and concomitant mitral valve endocarditis with large vegetation.

The patient was taken to the operating theater the next day. We performed a median incision and drained the mediastinal abscess, debrided her fractured sternum, including the cartilaginous portions of the transverse second and third ribs, the left internal mammary artery and the left pectoralis muscles with necrotic subcutaneous tissues. The manubrium of the sternum was spared from debridement since there was no gross evidence of infection, leaving a 12-cm defect in the mid-portion of the sternal body. After the debridement, vacuum-assisted closure (VAC) therapy with continuous saline washing was performed. The first wound culture demonstrated the presence of Methicillin-sensitive *S. aureus* (MSSA), and the antibiogram was identical to that of the MSSA isolate from the blood. Intravenous vancomycin was changed to intravenous cefazolin.

**Fig. 3:**
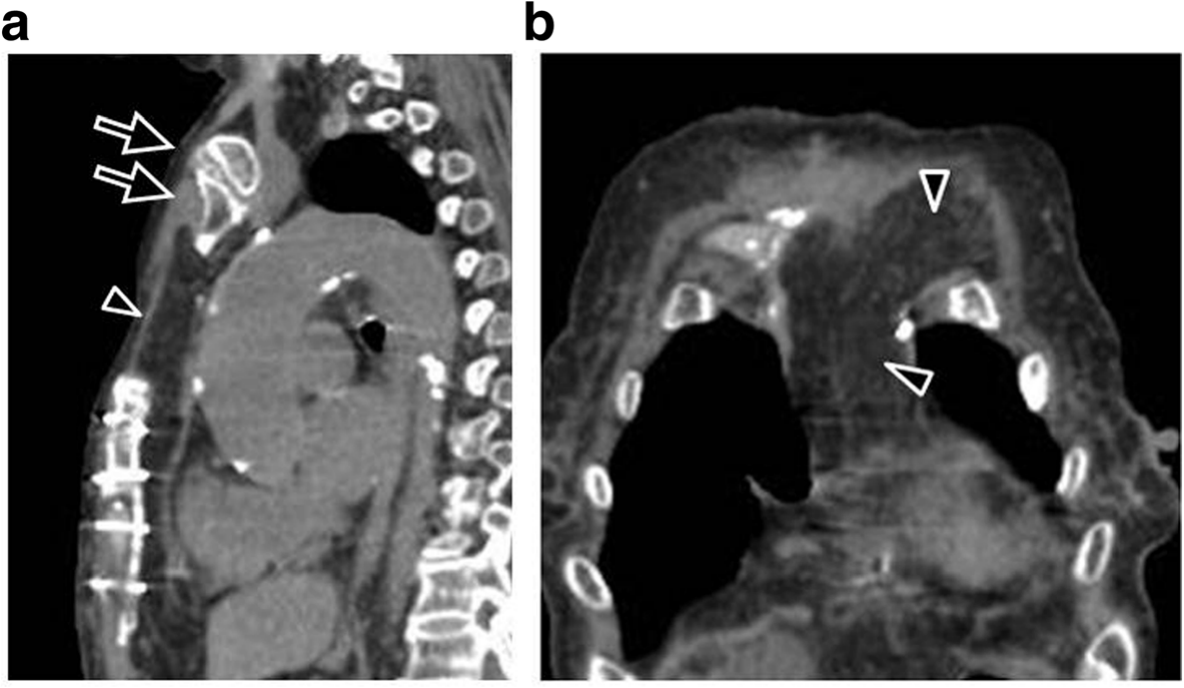
Contrast-enhanced computed tomography two months postoperatively. **a** Sagittal image. **b** Axial image. The manubrium of the sternum was intact (*arrows*) and a healthy omental flap covered the sternal defect (*arrowheads*). Postoperative computed tomography

Over the next week, she underwent two wound irrigation procedures. Although subsequent cultures at dressing changes were all negative in this period, she had continuously high grade fever and her laboratory data still demonstrated high. After extensive discussion of the benefits and risks of the surgical intervention because of the significant co-morbidities, we decided to perform the vegetectomy and mitral valve repair. The cardiopulmonary bypass was established through lower partial sternotomy. Surgery revealed perforation with a large vegetation of the posterior mitral leaflet, which was completely resected and successfully repaired with an autologous pericardial patch (Fig. 2b). The aortic crossclamp time was 98 min and cardiopulmonary bypass time was 132 min. After 4 days, the lower sternum was apposed using sternal wires and the chest wound was completely closed with an omental flap (Fig. 3a, b).

There were no pulmonary complications and the patient received intravenous cefazolin for 8 weeks. At the 8th month follow-up period, the patient showed no signs of infection and was doing well without any significant mitral insufficiency.

### Discussion

There are some reports of sternal fractures with osteomyelitis as a complication of closed chest cardiopulmonary resuscitation [3], and are not uncommon in the setting of antecedent chest wall trauma, such as thoracic surgery [4, 5]. However, a mediastinal abscess because of a primary traumatic sternal fracture is uncommon [1, 2]. Cuschieri et al. identified the presence of hematoma, intravenous drug use, and a source of staphylococcal infection as risk factors for developing a posttraumatic mediastinal abscess [6]. Of note, the majority of these patients have an underlying systemic disease such as diabetes mellitus, liver dysfunction, or compromised host [7]. In our case, the patient had no remarkable co-morbidity, experienced only a traumatic episode 1 month earlier and she was accompanied by the native valve endocarditis. To the best of our knowledge, this catastrophic situation is extremely rare and has not been previously reported, nor has a successful strategy been employed to manage this situation; hence, we described this case.

Once diagnosed this serious co-infected case, the primary concern of any surgical procedure is to control the bacteria infection [8]. Treatment of mediastinitis may depend on the degree severity of the infectious; conservative therapy for mild infections, or single staged intervention such as debridement and primary wound closure for moderate infections, or leaving the wound open and second-stage closure using the several closure techniques available for severe cases [9, 10]. Urbanski et al. reported a case of chronic sternal osteomyelitis after radiation and severe aortic valve stenosis [11]. The patient was treated simultaneously by sternal debridement and concomitant aortic valve replacement, and single-stage primary wound closure. This was a case of chronic non-severe mediastinitis; therefore, the single staged intervention could be performed. In our case, the mediastinal abscess was already extensive and progressed in a few days; furthermore, the patient had concomitant endocarditis with uncontrolled infection and considerable risks of embolism from the lerge vegetation. Thus, we decided on a staged strategy: first, open drainage for mediastinitis; second, cardiac operation for endocarditis; and finally, primary wound closure with omental flap.

It remains unclear when the endocarditis with the vegetation should be treated in this co-infected situation. For example, Kang et al. demonstrated that early surgical intervention in patients with large vegetation (a diameter greater than 10 mm) may significantly decrease embolic events and in-hospital mortality [12]. Moreover, the risk of embolism has been reported to be particularly high during the first week after diagnosis. However, cardiac operations performed too early had the disadvantage of increasing the risk for recurrent mediastinitis. In our patient, although she had VAC therapy for about 2 weeks and underwent four procedures from initial debridement to cardiac operation, the infection was not controllable because of the large vegetation. We thought this timing was best to perform the vegetectomy.

In treating the mitral valve endocarditis, several surgical approaches may be employed. The right thoracotomy approach could avoid going through the infected field and decrease the recurrence of the bacteria. However, the major disadvantage is the need for an additional right-sided thoracotomy incision, which requires a longer mechanical ventilation period, as is often the case with our older patients after aggressive debridement of the sternum. With partial sternotomy, Kaneda et al. reported the usefulness of a lower partial sternotomy for acutely infected endocarditis in patients with tracheostoma [13]. When the manubrium of the sternum could be spared from debridement, the lower partial sternotomy approach was associated with benefits, including thoracic stability because of preservation of the sternoclavicular joint and the upper ribs. In our case, we believe that lower partial sternotomy could be performed and sparing the manubrium of sternum led to a favorable result.

## Conclusions

In conclusion, this successful staged strategy has not been previously reported. We believe that our quick decision about repeated surgical interventions and preservation of the manubrium led to a favorable result.

## Abbreviations

VAC, vacuum-assisted closure therapy; CT scan, computed tomographic scan
